# Making Sense of Electrical Stimulation: A Meta-analysis for Wound Healing

**DOI:** 10.1007/s10439-023-03371-2

**Published:** 2023-09-24

**Authors:** Mamun Rabbani, Enayetur Rahman, Michael B. Powner, Iasonas F. Triantis

**Affiliations:** 1 Research Centre for Biomedical Engineering, School of Science and Technology, City University of London, Northampton Square, London, ECIV 0HB UK; 2 Centre for Applied Vision Research, School of Health and Psychological Sciences, City University of London, Northampton Square, London, ECIV 0HB UK

**Keywords:** Stimulation protocol, Wound healing, Electro-modulation, Skin wounds, Impedivity, Current of injury, Trans-epithelial potential, Electrodes

## Abstract

Electrical stimulation as a mode of external enhancement factor in wound healing has been explored widely. It has proven to have multidimensional effects in wound healing including antibacterial, galvanotaxis, growth factor secretion, proliferation, transdifferentiation, angiogenesis, etc. Despite such vast exploration, this modality has not yet been established as an accepted method for treatment. This article reviews and analyzes the approaches of using electrical stimulation to modulate wound healing and discusses the incoherence in approaches towards reporting the effect of stimulation on the healing process. The analysis starts by discussing various processes adapted in in vitro, in vivo, and clinical practices. Later it is focused on in vitro approaches directed to various stages of wound healing. Based on the analysis, a protocol is put forward for reporting in vitro works in such a way that the outcomes of the experiment are replicable and scalable in other setups. This work proposes a ground of unification for all the in vitro approaches in a more sensible manner, which can be further explored for translating in vitro approaches to complex tissue stimulation to establish electrical stimulation as a controlled clinical method for modulating wound healing.

## Introduction

Skin wounds have been one of the prime causes of hospitalization in the past few decades. Various studies show the ever-increasing expense in healthcare due to wounds [1, 2]. The natural wound healing mechanism of the skin is a structured and timely process that starts from the time of wounding and may persist for a period of time, which depends on the type of wound [3]. This orderly process might get disrupted due to various underlying comorbidities [4, 5]. Prolongation of the healing procedure results in physical and mental trauma of the patient and also creates an overhead cost for the healthcare system. Research has been going on for a while to shorten the duration of healing of skin wounds.

**Fig. 1:**
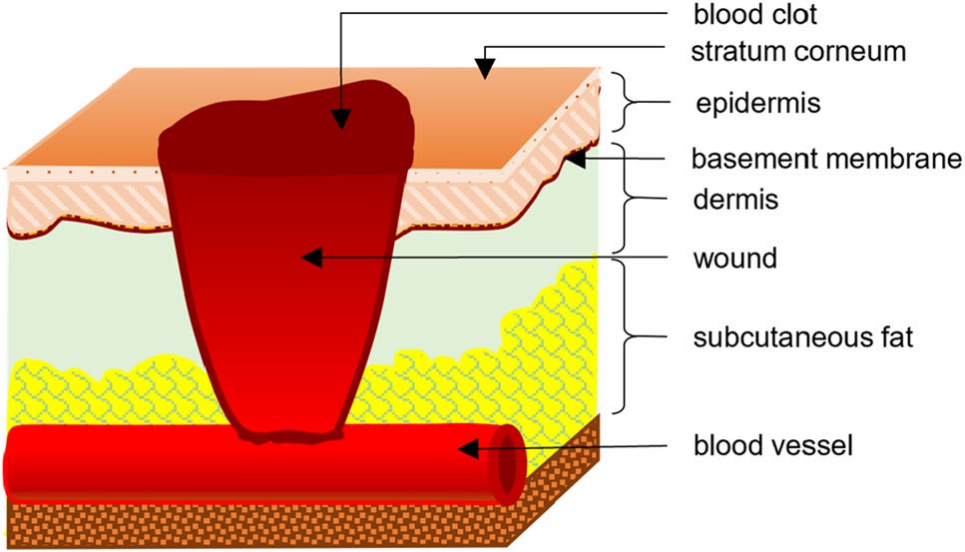
Schematic anatomy of skin wound showing different tissue layers

**Fig. 2:**
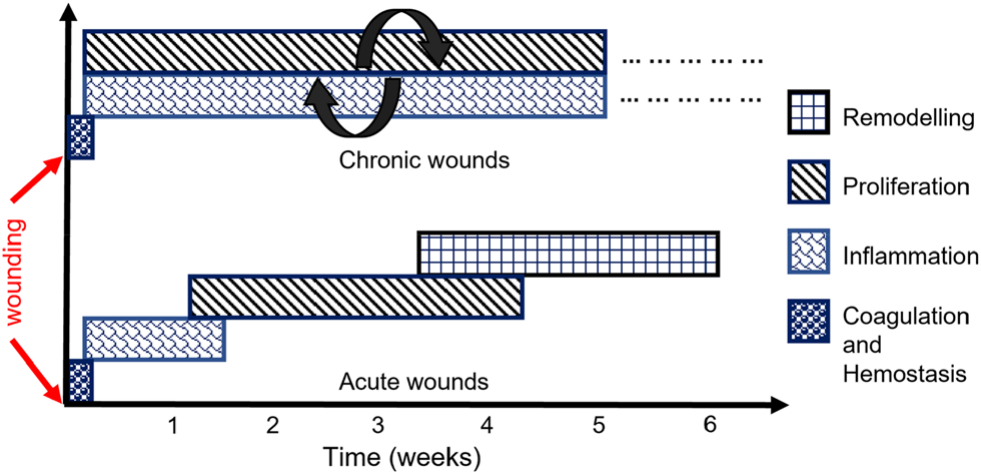
Comparison between acute and chronic wound healing [8, 10]

Any injury or disorder in the natural anatomy of the skin ranging from a break in the epithelial structure of the skin up to damage in deep subcutaneous tissues and organs is defined as a wound [6, 7] (Fig. 1). In response to wounding, the body initiates a series of physiological mechanisms to isolate, disinfect, regrow, and heal the affected tissue within an expected course of time, known as wound healing [3]. Time frame is an important factor in wound healing. A timeline of 4 to 6 weeks is generally observed for the healing process [8]. Wounds that heal in an orderly manner within this expected timeline resulting in an anatomically balanced and functional tissue are known as Acute wounds [3, 7]. While some wounds fail to heal either within the expected time frame or in the expected manner, such wounds are defined as chronic wounds [7, 9]. Usually chronic wounds are identified by prolonged inflammation persisting over months and not being healed for years [10, 11]. Several underlying factors such as hypoxia, bacterial infection, lack of blood perfusion, and change in cellular response lead to an impaired healing process which results in chronic wounds [8]. The process of natural wound healing can be distinguished into four stages based on the underlying physiological mechanisms [12]. For acute wounds, the stages of healing are maintained in an orderly manner within the prescribed timeline, whereas for chronic wounds, the inflammation stage is prolonged, the outcomes are uncoordinated, and result in poor healing. A comparison between the healing timeline of acute wounds and chronic wounds is shown in Fig. 2, where it can be seen that the healing stages for acute wounds are in an orderly manner within the expected timeline of around 6 weeks [8], whereas for chronic wounds, the inflammatory and proliferation stage continues in parallel. The persisting inflammation breaks down the proliferated cells resulting in incomplete healing of the wound.

**Fig. 3:**
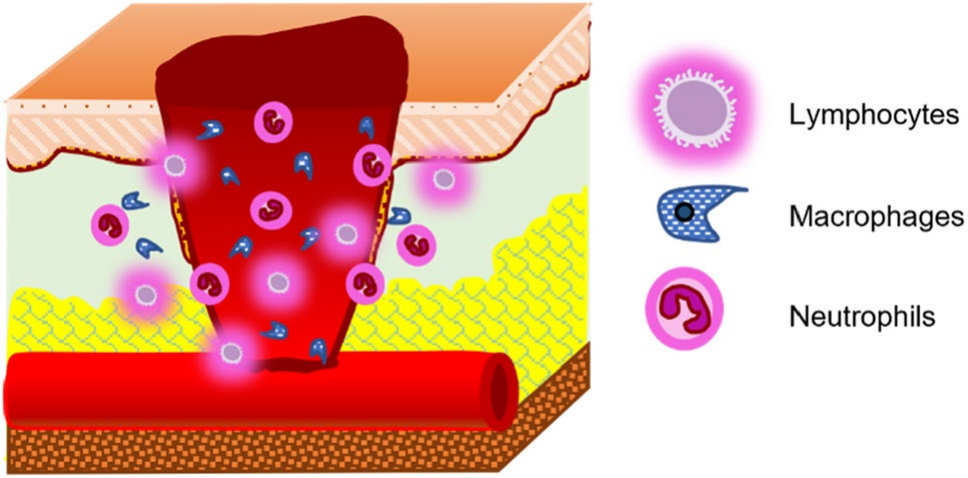
Inflammatory stage of wound healing

**Fig. 4:**
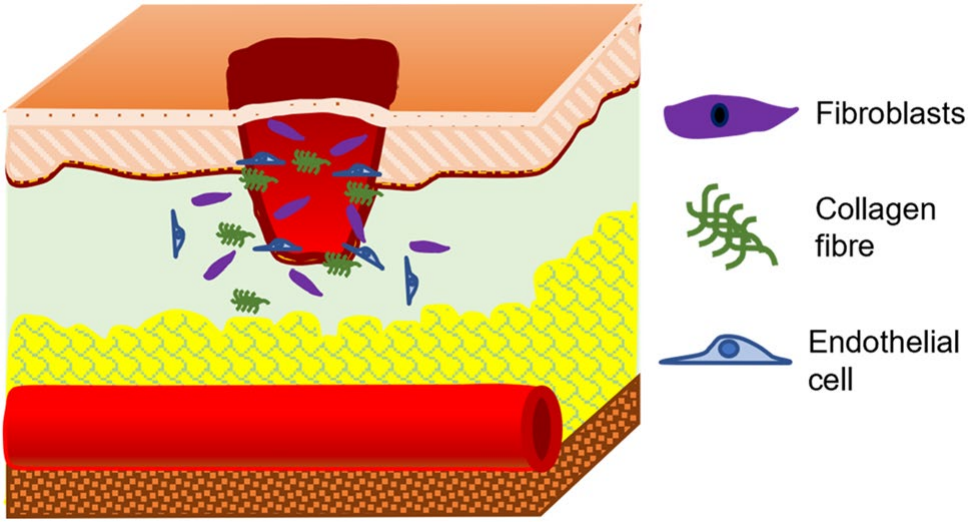
Proliferative stage of wound healing

**Fig. 5:**
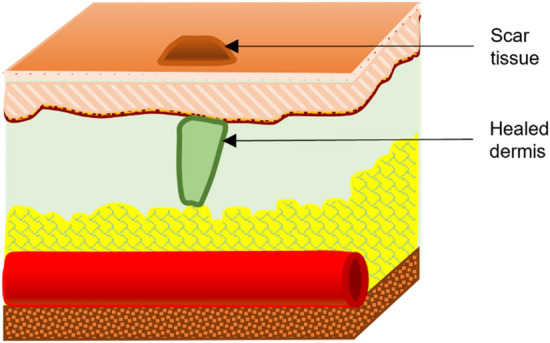
Remodeling stage of wound healing

The natural wound healing process is complex and coordinated involving various biological and immunological systems, where different mechanisms tend to curate the wound site in various ways [13, 14]. The first stage of healing is known as *Coagulation and Hemostasis*, which targets limiting blood outflow from wounds by making a clot [7, 15, 16]. Several clotting factors and growth factors are secreted in this stage which are essential in the later stages of healing [7, 15–18]. The second stage of healing, known as *Inflammation* (Fig. 3), immunizes the wound site from microorganisms [19]. Phagocytosis of foreign bodies by neutrophils and debridement of the wound site by macrophages and lymphocytes takes place along with activation of keratinocytes, fibroblasts, and endothelial cells [19–22]. *Proliferation* is the third stage of healing (Fig. 4), where tissue repair is initiated by migration of growth cells and development of vascular networks [12, 19]. Fibroblasts generate new extracellular matrix [22], collagen provides structural integrity [23], endothelial cells protrude the wound site to create new microvascular networks [24], and granular tissues produce scar at the site [25]. The final stage is called *Remodeling* (Fig. 5), where the new epithelium is created replacing the initial epithelium generated during hemostasis and formation of the final scar [21, 26].

Over the years, there have been reports of using chemical, electrical, optical, and magnetic means to modulate the wound healing process [27–30]. Further advancements have also been reported based on the type of wounds [31]. Among all the reported modalities for enhancing wound healing, electrical stimulation has been the most widely explored method. The earliest instances of electrical stimulation involved induced wounds in animal models [32]. The results showed great improvement in various sorts of wounds ranging from burn to chronic wounds [33, 34]. Researchers have also explored the effect of electrical stimulation on particular cell types in vitro, aiming to enhance specific stages of healing [35–38]. Many groups have also conducted clinical trials to demonstrate the efficacy of electrical stimulation in wound healing [39–42].

Despite being widely explored, there are inconsistencies in reporting the parameters of stimulation. The different electrical phenomena seen in biological mediums due to different stimulating circuitry and setups present complexity and dependence of the applied stimulus on various parameters. The absence of reporting any one of the parameters puts forth the issue of repeatability of the method. A reported method might not be completely realized and applied for further research if it lacks repeatability due to a lack of information about parameters. Scalability of stimulus helps in designing methods for various sizes of wounds and can be achieved by incorporating experimental and setup parameters along with the value of the stimulus applied.

Effects of various stimulation parameters on excitable tissues have been reported [43]. A protocol for reporting electrical stimulation on excitable tissues is also established [44]. A similar methodological protocol for reporting electrical stimulation in wound healing would be instrumental in providing a pathway for connecting various approaches.

This work analyzes different approaches to electrical stimulation for wound healing. Various parameters involved in modulating the electrical stimulus are discussed and their effects on the repeatability and scalability of experiments are highlighted. This work also emphasizes the need for a standardized protocol in reporting electrical stimulation and recommends a protocol to make sense of electrical stimulations in wound healing.

## Physiological Factors in Wound Healing

**Fig. 6:**
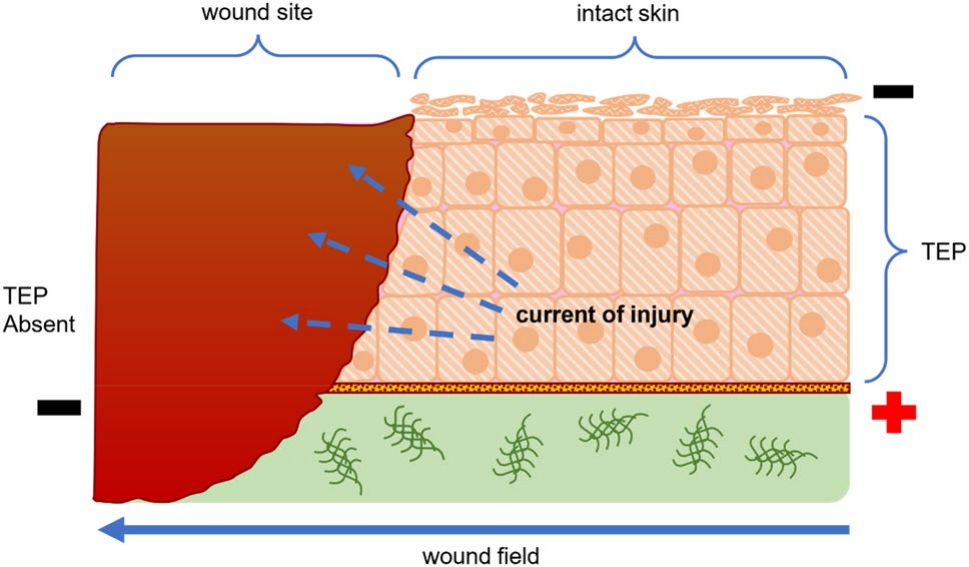
Trans-epithelial potential (TEP) and current of injury: intact skin vs. wound site

Tissues have a potential gradient across their epithelial layer, known as the trans epithelial potential (TEP) [45, 46]. It is due to the resistive nature of the epithelial layer keeping minimal flow of ions across the concentration gradient. The value of TEP is around 10–60 mV/mm, being more positive on the inside of the tissue compared to the outside, as shown in Fig. 6 [45, 46]. The potential across the epithelium is responsible for various physiological mechanisms, like maintaining the transparency and balance of water in corneal tissue (non-excitable) or causing rhythmic pulses in cardiac tissues (excitable).

In the case of a skin injury, the highly resistive epithelial layer is distorted, disrupting the TEP and giving rise to a potential difference at the wound site. An electric field gradient of around 150–200 mV/mm is generated towards the wound site, resulting in a current flow towards the wound known as *Current of injury* [47, 48]. This endogenous current, ionic in nature, is a natural response of the body and modulates the healing process. Animal models have reported a current of injury of around 3 µA/cm^2^ [49]. Various works have reported a positive correlation between ion movements (Na^+^, Cl^−^, K^+^, Ca^2+^) into the wound site and the current of injury [50–52]. Vieira et al. showed in in vitro experiments that an increase in Cl^−^ regulation causes the current of injury to rise [50]. In vivo studies on newt limb regeneration have shown a presence of current of injury in amputated limbs which are dependent on ionic concentration at the wound [53, 54]. It is also been reported that an external flow of current through the wounded limb enhanced growth [55]. An interesting work by Shen et al. showed that a weaker current of injury results in delayed healing of diabetic corneal wound [56]. A proportionality between enhancing the current of injury and wound healing can be drawn. Hence, any external stimulation enhancing specific ion concentrations at the wound site or bolstering the endogenous current would positively affect wound healing.

Movement of various cells towards the wound site is observed in various stages of wound healing. Cells demonstrate different directional affinities under the influence of electric fields. The electric fields generated due to the current of injury at the wound site provide a directional cue for the specialized cells to migrate to the site and initiate the healing process. Such directional migration of cells is known as *Galvanotaxis*. Kloth summarized the polarities of cells involved in wound healing [57]. Different works have reported directional taxis of various cells due to external stimulations including fibroblast [47], keratinocytes [38], lymphocytes [58], vascular endothelial cells [59] which are critical in different stages of wound healing. Hence any external electrical stimulation would promote the directional migration of cells and enhance the wound healing process.

*Growth factors* are an integral part of wound healing as they contribute to the proliferation and transdifferentiation of cells. Different growth factors like EGF, PDGF, TGF-β, FGF, VEGF, etc. play vital roles in the stages of wound healing. Works from various research groups show a prominent effect of electrical stimulation on the growth factors. Regulations and modulations have also been reported for EGF [60], VEGF [61], PDGF [62], TGF-β [63], and FGF [64]. A recent in vitro study by Cui et al. reported the upregulation of IL-6, IL-1α, IL-8, GROα, FGF2, and VEGF-A in human skin models due to application of external electric fields [65]. A similar result has also been reported by Urabe et al. where applying pulsed electric stimulation increased PDGF-A, FGF2, and TGF-β1 attributing in human dermal fibroblast proliferation [66]. Therefore, it is safe to say that electrical stimulations can be used to enhance the healing process of wounds by modulating growth factors.

From the discussion above, it is evident that any external electric stimulation enhances the current of injury, provides directional cues for migratory cells, and upregulates growth factors for cell proliferation in the wound site accelerating the process of wound healing.

## Overview of Approaches

Electrical stimulation has been widely explored in influencing both excitable and non-excitable tissues. Various research groups have shown the effect of electrical stimulation in tissues like bone, muscle, nerve, cardiac, etc. [67–70], as well as at the cellular level [71]. Not hard to say that electrical stimulation has also been explored to accelerate the process of wound healing. Different stimulation parameters used by various groups in the form of current, voltage, and electric field are discussed in the following sections. The literature can be analyzed according to the type of experiments (in vitro, in vivo, clinical), in terms of stimulation parameters (DC, pulsed DC, AC), and in terms of affecting the healing process (inflammation, proliferation, remodeling).

### Current Stimulation

#### In Vitro

In vitro experiments using current as a stimulating parameter have been summarized in Table 1. Effects on the inflammatory stage of wound healing have been observed with DC stimulation. These effects range from bactericidal [72], bacteriostatic [73], inhibition of growth [74–76], and decreasing the number of viable bacteria [35]. The amplitude of the DC stimulation is mostly observed in the range of 0.4 µA to 1 mA although del Pozo et al. applied a significantly higher amplitude of direct current to decrease the number of viable bacteria [35]. Pulsed current stimulation of 6000 ppm and 3–9 mA intensity has been used by Gomes et al. for inhibition of bacterial growth [76].

**Table 1:** Current stimulation for wound healing, in vitro experiments

Stimulation parameters			Electrode parameters			Effect of stimulation		References
Type	Amplitude	Frequency (ppm)	Duration	Material	Dimension	Healing phase	Effect	
DC	0.4–400 µA		48 h	SS, Pt, Au, Ag	20 mm length, 25 mm apart	Inflammatory	Bactericidal effect on *S. aureus*	[72]
DC	0.4–400 µA			SS, Pt, Au, Ag, Cu	20 mm length, 0.2–0.4 mm apart	Inflammatory	Bacteriostatic effect and inhibited growth of bacteria	[73]
DC	0.2–1 mA		2–18 h	Platinum iridium	2 cm length	Inflammatory	Inhibited growth of *C. albicans*	[74]
DC	20–2000 mA		1–7 days	SS or graphite	55 mm length	Inflammatory	Decreases the number of viable bacteria	[35]
DC	500 µA		1 h	SS wire	1.2 cm length, 1.5 cm apart	Inflammatory	Inhibited growth of bacteria	[75]
Pulsed DC	3, 6, 9 mA	6000	15, 30 min	SS semicircular	10 cm^2^	Inflammatory	Inhibited growth of bacteria	[76]
DC	2 µA			Spray electrode	3 mm thick (approx.)	Proliferative	Promoted growth and migration of fibroblast	[77]
Pulsed DC	50 µA	6000	20 min	SS wires	$2.2 \times 1.5$ cm^2^, 7.5 cm apart	Proliferative	DNA synthesis for fibroblast proliferation	[80]
Pulsed DC	0, 50, 100 µA	18	4 h	Platinum	20 × 5 mm, 35 mm apart	Proliferative	Galvanotaxis of fibroblasts	[78]
Pulsed DC	2–100 µA	11	3 Times every 5 h	Triboelectric nanogenerator	100 nm diameter, 1 µm length	Proliferative	Modulated the growth of fibroblasts	[79]

Different in vitro experiments also showed an effect of the proliferative stage of wound healing. Various stimulation primarily affected the directional migration of fibroblasts [36, 77, 78], promoted growth of fibroblasts [77, 79], and enhanced DNA synthesis for fibroblast proliferation [80]. Konstantinou et al. applied a low direct current of 2 µA, promoting the growth and migration of fibroblasts [77]. Whereas Snyder et al. went up to 18.24 mA of direct current corresponding to 100 mV/mm constant electric field across the in vitro samples showing random migration of fibroblasts [36]. Bourguignon et al. explored pulsed DC stimulations with an amplitude of 50 µA and pulse rate of 6000 ppm to enhance DNA synthesis for fibroblast proliferation [80]. Although, a couple of other works applied pulsed DC with a higher intensity of around 50–100 µA with a lower pulse rate of 11–18 ppm [78, 79].

#### In Vivo and Ex Vivo

A wide range of in vivo and ex vivo experiments portraying the effect of stimulating current on wound healing are briefed in Table 2. DC stimulations in vivo have been shown to shorten the duration of the inflammation stage [64, 81–83], bacteriostatic effect [32], recruitment of immunocytes and cytokines [84] and reduction of mast cells [82]. Ex vivo stimulation on human tissues showed bactericidal effects [85] and reduction of mast cells [86] to enhance the inflammatory stage of wound healing. Stimulations from as low as 300 µA up to 1 mA have been reported by various groups to show an effect on the inflammatory processes [32, 81]. Although in the case of human skin tissues, the amplitude is found to be much lower, around 10–100 µA, showing a bactericidal effect [85]. Asadi et al. explored the effect of both DC and pulsed DC stimulations on rat tissues reporting the same effect for both types of stimulation. They showed that a DC intensity 600 µA and a pulsed DC of 2.5–3 mA with 6000 ppm modulated FGF2 levels and shortened the inflammation stage in separate in vivo experiments with rat tissue [64]. Other groups have also explored the effect of pulsed DC on inflammation of wounds with stimulation parameters ranging from 300 µA to 40 mA and pulse rates within 30–120 ppm [83, 84]. Interestingly, Reich et al. and Weiss et al. used similar stimulation parameters of 35 mA and 7680 ppm to reduce mast cells in separate experiments with pig tissues and human tissues respectively [82, 86]. Various effects of current stimulation on the proliferation stage of wound healing have been reported in many works which include increase of fibroblasts [87–90], collagen secretion [87, 91, 92], migration of epithelial cells [91], release of VEGF [92, 93], decreasing PMN [94], increasing blood vessels [89] and increasing tensile strength to enhance wound closure [88, 90]. Effects on the final stage of wound healing, such as reduction of wound area [95, 96], are also explored in a few works. DC stimulations are seen to have ranged from 20 to 300 µA in different works involving the growth and proliferation of fibroblasts [87, 94]. A slightly higher current intensity is seen for pulsed DC stimulations, around 300–600 µA and 4800 ppm [90]. Although Morris et al. used a higher current of 11 mA and lower pulse rate of 1500 ppm to enhance proliferative mechanisms [92]. Asadi et al. used both DC stimulation of 600 µA and pulsed DC stimulation of 2.5–3 mA with 6000 ppm in separate experiments to enhance the release of VEGF [93]. Reger et al. also experimented with two different stimulations separately, 0.6 mA DC and 7–10 mA with 2400 ppm pulsed DC, resulting in reduced wound area in both cases [95]. The discontinuous nature of pulsed DC demands higher current intensity than the DC counterpart. In a different approach, Borba et al. used AC stimulation of 8 mA and 7.7 Hz to increase fibroblast and blood vessels in rabbit tissue [89].

**Table 2:** Current stimulation for wound healing, in vivo and ex vivo experiments

Stimulation parameters			Electrode parameters			Effect of stimulation		References
Type	Amplitude	Frequency (ppm)	Duration	Material	Dimension	Healing phase	Effect	
DC	300 µA		30 min/day	Carbonized rubber		Inflammatory	Shorten the inflammatory phase in rat tissue	[81]
DC	600 µA		1 h/day	Carbonized rubber	1 × 3 cm^2^ active, 2 × 4 cm^2^ passive	Inflammatory	Modulates FGF2 levels and shortens the inflammation phase in rat tissue	[64]
Pulsed DC	2.5–3 mA	6000	1 h/day	Carbonized rubber	1 × 3 cm^2^ active, 2 × 4 cm^2^ passive	Inflammatory	Modulates FGF2 levels and shortens the inflammation phase in rat tissue	[64]
DC	1 mA		7 h	Cu mesh	1.5 × 2 in.^2^	Inflammatory	Bacteriostatic effect on rabbit	[32]
DC	10–100 µA		4–24 h	Carbonized circular electrodes	10 cm^2,^ 10–15 cm apart	Inflammatory	Bactericidal effect on intact human skin	[85]
Pulsed DC	300–600 µA	0.138–30	1 h	PDMS–PEDOT:PSS	3 mm diameter, 9 mm apart	Inflammatory	Recruitment of immunocytes and cytokines in mouse tissue	[84]
Pulsed DC	35 mA	7680	30 min, twice everyday	Sponge electrode	6 × 6 cm^2^	Inflammatory	Reduce mast cell and shorten inflammation in pig tissue	[82]
Pulsed DC	40 mA	120	15 min/day	Carbonized silicon rubber	2 × 1.5 cm^2^, 1.5 cm apart	Inflammatory	Reduce pro-inflammatory cytokines and shorten inflammation in rat tissue	[83]
Pulsed DC	35 mA	7680	30 min, twice everyday	Saline-soaked cellulose pad		Inflammatory	Reduce mast cells and scars in human tissue	[86]
DC	50–300 µA		24 h	Ag-coated nylon	7 × 10 mm^2^ treatment area	Proliferative	Migration and proliferation of epithelial cells and collagen secretion in pig tissue	[91]
DC	20–100 µA		30 min, twice everyday	Carbon-fiber electrode on collage sponge	12 × 8 cm^2^	Proliferative	Increased fibroblast and collagen fiber in pig tissue	[87]
DC	600 µA		1 h/day for 2 days	Carbonized rubber	1 × 3 cm^2^ active, 2 × 4 cm^2^ passive	Proliferative	Release of VEGF in rat tissue	[93]
Pulsed DC	2.5–3 mA	6000 ppm	1 h/day for 2 days	Carbonized rubber	1 × 3 cm^2^ active, 2 × 4 cm^2^ passive	Proliferative	Release of VEGF in rat tissue	[93]
DC	300 µA		30 min	Saline-soaked carbonized rubber		Proliferative	Decrease PMN, increase fibroblast in rat tissue	[94]
Pulsed DC	300–600 µA	4800 ppm	1 h for 21 days	Carbonized rubber	2 × 3 cm^2^, 3 cm apart	Proliferative	Enhances wound closure, increases fibroblast and collagen formation	[90]
Pulsed DC	11 mA	1500 ppm	14 days			Proliferative	Increases secretion of VEGF and collagen in rabbit tissue	[92]
AC	8 mA	7.7 Hz	30 min for 14 days	Saline-soaked aluminum electrodes	5 × 3 cm^2^, opposite side of animal body	Proliferative	Increase fibroblast and blood vessel in rabbit tissue	[89]
DC	0.6 mA		2 h/day, 5 days/week for 30 days			Remodeling	Reduction of wound area	[95]
Pulsed DC	7–10 mA	2400 ppm	2 h/day, 5 days/week for 30 days			Remodeling	Reduction of wound volume	[95]

#### Clinical

Various clinical trials for current stimulation in wound healing are summarized in Table 3. Stimulations have proven to be effective in wound area reduction for chronic wounds [34, 97–100], pressure wounds [101–103], diabetic wounds [39, 104], and venous ulcers [40]. Other works also report an increase in angiogenic response and increasing levels of hemoglobin in acute wounds [105, 106]. The magnitude of DC stimulation ranges from 200 to 800 µA, contributing to a reduction in wound area [97, 101]. Wirsing et al. reported that a low-intensity DC stimulation of 1.5 µA applied through a wireless microcurrent stimulator also contributes to area reduction in chronic ulcers [34]. Pulsed DC stimulations are also seen to decrease the wound area in different sorts of wounds, having an amplitude quite similar to that of DC stimulations with pulse rates up to 6000 ppm [102, 103]. Feedar et al. lowered the amplitude of pulsed DC to 29.2 µA applying a high pulse up to 7680 ppm to achieve healing in chronic wounds [98]. A few groups experimented with AC stimulations in the mA range with frequencies around 30–60 Hz resulting in improving angiogenic response and wound area reduction [39, 106]. Frequencies of 1 kHz are also seen for some specific mechanisms involving localized stimulations [40, 99, 100, 104].

**Table 3:** Current stimulation for wound healing, clinical experiments

Stimulation parameters			Electrode parameters			Effect of stimulation	References
Type	Amplitude	Frequency (ppm)	Duration	Material	Dimension	Effect	
DC	1.5 µA		45–60 min, 2–3 times/week	Wireless microcurrent stimulator		Wound area reduction in chronic ulcers	[34]
DC	300–500 µA, 500–700 µA		2 h for 5 days	SS mesh	15–25 cm apart	Wound area reduction in chronic ulcers	[97]
DC	200–800 µA		2 h, 3 times/day, 4 weeks		25 cm apart	Wound area reduction in pressure ulcers	[101]
Pulsed DC	300–600 µA	48 ppm	3 times/week		2 cm around wound	Wound area reduction and closure of pressure ulcers	[102]
Pulsed DC	29.2 µA	3840–7680 ppm	30 min, twice everyday	Sponge covered SS electrode	7.5 × 7.5 cm^2^ active, 16 × 16 cm^2^ passive	Wound area reduction in chronic ulcers	[98]
Pulsed DC	500 µA	6000 ppm	1 h for 20 days	Aluminum	20 × 25 cm^2^	Wound area reduction in pressure ulcers	[103]
AC	200 mA	30 Hz	3 times/week		2 × 4 in^2^ bipolar	Wound area reduction in diabetic wounds	[39]
AC	100–170 µA	1–1000 Hz	40 min, 5 days/week for 21 days		4 Pairs of electrodes, 1 cm^2^ contact area	Wound area reduction in chronic ulcers	[99]
AC	100–170 µA	1–1000 Hz	3–4 times/week		4 Pairs of electrodes, 1 cm^2^ contact area	Wound area reduction in chronic ulcers	[100]
AC	100–170 µA	1–1000 Hz	30 min every 2 days for 30 days		4 Pairs of electrodes, 1 cm^2^ contact area	Wound area reduction in diabetic foot ulcers	[104]
AC	100–170 µA	1–1000 Hz	25 min, 5 days/week for 21 days		4 Pairs of electrodes, 1 cm^2^ contact area	Wound area reduction in venous ulcers	[40]
AC	0.004 mA	60 Hz	4 times/week for 90 days			Increasing angiogenic response in acute wounds	[106]

### Voltage Stimulation

#### In Vitro

The application of electrical stimulation in the form of voltage and electric field for wound healing has been reported in various literature. In vitro works involving voltage stimulation have been summarized in Table 4. Effects have been monitored in the stages of inflammation, proliferation, and remodeling. Voltage stimuli in the inflammation stage are shown to have antimicrobial effects and inhibit the growth of bacteria to maintain a favorable environment for healing [75, 76, 107, 108]. Electrotaxis of macrophages is also observed in DC voltage stimulations [109, 110] along with upregulation of hormones like TGFβ1 and ERK with pulsed DC, required in the later stages of wound healing, are also reported [111].

**Table 4:** Voltage stimulation for wound healing, in vitro experiments

Stimulation parameters			Electrode parameters			Effect of stimulation		References
Type	Amplitude	Frequency (ppm)	Duration	Material	Dimension	Healing phase	Effect	
DC	5–450 mV/mm		2 h	Ag/AgCl in agar salt bridge	50 mm apart	Inflammation	Electrotaxis of human macrophages towards cathode	[109]
DC	250 V		2 h	SS	0.035 Gage, 50 mm apart	Inflammation	Inhibits growth of *S. aureus, E. coli* and *P. aeruginosa*	[108]
DC	500 V		30 min	SS	5 mm diameter, 3 cm apart	Inflammation	Antimicrobial effects	[107]
Pulsed DC	100 mV/mm	3–6 ppm	24 h	Ppy-PET fabric		Inflammation	Upregulation of TGFβ1, ERK	[111]
Pulsed DC	32, 64, 95 V	6000 ppm	30–60 min	SS	10 cm^2^, semicircular	Inflammation	Inhibits bacterial growth	[76]
Pulsed DC	250 V	6000 ppm	1 h	SS wire	1.2 cm, 1.5 cm apart	Inflammation	Inhibits growth of *S. aureus*	[75]
AC	200 mV/mm	1 Hz	90 min	Pt	60 × 10 × 0.2 mm^3^ chamber	Inflammation	Migration of macrophages perpendicular to the electric field	[110]
DC	75–100 mV/mm			Ag/AgCl in agar salt bridge	22 mm apart	Proliferation	Promoted endothelial cells migration and release of VEGF in HUVEC	[61]
DC	50, 200 mV/mm		2–6 h	Ppy/HE/PLLA membrane	35 mm diameter	Proliferation	Increased proliferation of fibroblast, upregulated FGF1 and FGF2 secretion	[117]
DC	20–100 mV/mm			Ppy/HE/PLLA membrane		Proliferation	Increased fibroblast growth, decreased secretion of cytokine and growth factors	[118]
DC	100 mV/mm		24 h	Ppy/PLLA	2 cm apart	Proliferation	Increased cell viability in human skin fibroblast	[37]
DC	10–100 mV/mm				45–50 mm apart	Proliferation	Migration of keratinocytes	[38]
DC	100–200 mV/mm		6–24 h	Ppy-PU/PLLA		Proliferation	Proliferation of keratinocytes, secretion of cytokines and growth factors	[65]
DC	100–400 V/m			AgCl		Proliferation	Galvanotaxis of NIH-3T3 and SV101 cells	[114]
DC	50 mV/mm		3 h	PCL scaffold	1.5 × 1.5 cm^2^	Proliferation	Increased transdifferentiation in human dermal fibroblast	[123]
DC	50–200 mV/mm		6–18 h	Ppy/HE/PLLA membrane	4 cm^2^ surface area	Proliferation	Activated fibroblasts with keratinocytes	[119]
DC	100 mV/mm		2–5 h			Proliferation	Migration of fibroblast and effect on Golgi polarization	[112]
DC	0–100 mV/mm		3 h	Gold patterned electrodes	20 mm apart	Proliferation	Directional migration of neonatal human dermal fibroblasts	[115]
DC	25, 50, 100 mV/mm		> 10 min	Ag/AgCl in agar salt bridge	50 mm apart	Proliferation	Random migration of fibroblast	[36]
DC	300 mV/mm		3 h	Ag/AgCl in agar salt bridge	40 × 10 × 0.3 mm^3^ chamber	Proliferation	Golgi polarization and directional migration of CHO cells	[159]
DC	100–250 mV/mm		24 h	Ag/AgCl in agar salt bridge		Proliferation	Regulates growth factors for angiogenesis in HUVEC cells	[131]
DC	100–250 mV/mm		5 h	Ag/AgCl in agar salt bridge		Proliferation	Migration of epithelial cells in bovine corneal cells	[125]
DC	150–400 mV/mm		24 h	Ag/AgCl in agar salt bridge		Proliferation	Directional migration, reorientation, and elongation of vascular cells	[128]
Pulsed DC	0–300 V	6000 ppm	20 min	SS	2.2 × 1.5 cm^2^, 7 cm apart	Proliferation	Modulates protein and DNA synthesis in human fibroblast cells	[80]
Pulsed DC	0–10 V	6–600 ppm	0.5–24 h	Platinum		Proliferation	Increase expression of collagen, elastin, and collagenase in human dermal fibroblast	[130]
Pulsed DC	1–5 V	4800 ppm	15–60 min	Carbon		Proliferation	Increased growth in human dermal fibroblast	[66]
Pulsed DC	50, 100 mV/mm	3–6 ppm	24 h	Ppy-PET fabric		Proliferation	Improved cell migration, increase FGF2 secretion in human dermal fibroblast	[116]
Pulsed DC	0–300 V	6000 ppm	10 min	SS	2.2 × 1.5 cm^2^, 7 cm apart	Proliferation	Increases intracellular Ca^2+^ and insulin receptors	[160]
Pulsed DC	3–5 V	4800 ppm	5 min/day	Carbon silicon	3 cm apart	Proliferation	Keratinocyte differentiation with inhibited growth	[124]
AC	180 mV	1.688 Hz	1 h, twice/day	Carbon	3 mm thick, 18 mm apart	Proliferation	Promoted fibroblast growth and production of collagen in NIH-3T3 cells.	[120]
AC	2 V	1 Hz	24 h			Proliferation	Migration of fibroblast in L929 cells	[113]
AC	10 V	4 Hz	6–20 h	Biocompatible elastomeric patch		Proliferation	Proliferation and migration of human dermal fibroblast	[121]
AC	0.5 V	1 Hz	2 h, 3 times/day	Aluminum tapes	80 µm thick	Proliferation	Proliferation and migration of fibroblast in NIH-3T3 cells	[122]
AC	20–150 mV/mm	10–60 Hz	12 h	Ag/AgCl in agar salt bridge	44 × 13 × 11 mm^3^ chamber	Proliferation	Regulation collagen expression and cytotoxicity in human dermal fibroblast	[129]
DC	50–400 mV/mm		6 h			Proliferation	Electrotaxis of rat epidermal stem cells towards cathode	[126]
DC	25–150 mV/mm		3 h	Ag/AgCl in agar salt bridge	2 × 1 cm^2^ chamber	Proliferation	Electrotaxis of corneal epidermal cells	[127]

For the proliferation stage of wound healing, voltage stimulation has been reported to show effects on multiple mechanisms. For fibroblasts, stimulus has been shown to affect the migration [112–116], proliferation [66, 117–122] and transdifferentiation [123]. A similar effect of stimulation on the migration, proliferation, and differentiation of keratinocytes has also been documented [38, 65, 124]. Directional migration of cells towards the wound site, an important factor in wound healing, has also been shown to be affected by stimulations and various research have evidenced the migration of different cells including epithelial cells [125], endothelial cells [61], epidermal cells [126, 127] and vascular cells [128]. Such movement of cells due to stimulation gives a clear suggestion on the polarity of stimulus applied to the wound. Apart from the effect on specific cell types, electrical stimulation has also been seen to influence the secretion of cytokines [65, 118, 129], modulate protein and DNA synthesis [80], and increase the expression of collagen and elastin [120, 129, 130]. Besides, the upregulation of different growth factors like VEGF, FGF1, and FGF2 were also reported in literature [61, 65, 116, 117, 131].

The stimulation used in most of the works is applied as an electric field across samples, although a few works have been seen to apply voltages. A few groups have applied DC voltages [107, 108], while others applied pulsed DC voltages [75, 76], although their works were primarily involved with the inhibition of bacterial growth. Most works involving cell mechanisms and modulation of biological factors used an electric field as stimulation. The electric fields were applied in the form of DC, pulsed DC, or AC. The applied DC fields were around 450 mV/mm to support inflammatory stages [110] while it is observed to be around 150 mV/mm in experiments involving proliferation mechanisms [117, 119]. The pulsed stimulations have a rate of around 6000/min for both voltage and electric field applications [76, 80]. The groups working with AC stimulations also had a similar amplitude to DC and pulsed DC configurations, with a frequency mostly under 5 Hz [110, 121] although there are instances of AC electric field stimulation having a frequency up to 60 Hz [129].

#### In Vivo and Clinical

The in vivo works of different groups are summarized in Table 5. The in vivo works reported modulation of different healing mechanisms including angiogenesis [132], epithelial formation [132, 133], upregulating of αSMA, and TGFβ1 [134], increasing tensile strength of wounds [135], inhibiting scar formation [132] and enhancing wound closures in animal models [136, 137]. Most of the in vivo experiments preferred using voltage stimulation reaching a value around 50 V and 6000 ppm. Although Liang et al. used pulsed electric field stimulation with a pulse rate of 1000 pps (60,000 ppm) which is quite high compared to other studies [133], Cinar et al. experimented with accelerating wound healing in mice tissues in vivo with a pulsed electric field of 900–1900 mV/mm and a pulse rate of 30 kHz ($60 \times 30 \times {10}^{3}$ ppm), which is extremely high compared to the stimulation parameters used in other studies [137].

**Table 5:** Voltage stimulation for wound healing, in vivo experiments

Stimulation parameters			Electrode parameters			Effect of stimulation		References
Type	Amplitude	Frequency (ppm)	Duration	Material	Dimension	Healing phase	Effect	
Pulsed DC	40 V	6000 ppm	1 h for 2 days			Proliferation	Increases angiogenesis and epithelial formation, inhibits scar in rat tissue	[132]
Pulsed DC	35–50 V	100 ppm	40 min for 1 week	Carbon–silicon rubber	2 × 2 cm^2^	Proliferation	Increases αSMA, TGFβ1 in diabetic rat wounds	[134]
Pulsed DC	20 V	30 ppm	5 days	Silicon rubber or carbon	1.5 cm^2^	Remodeling	Enhance wound closure rate	[136]
Pulsed DC	100 mV/mm	1000 ppm		Carbon fiber		Remodeling	Migration of epithelium and formation of new epithelium in pig wound	[133]
Pulsed DC	0–12.5 V	1200 ppm	15 min/2 days	Metallize gauze	2 cm^2^	Remodeling	Increases tensile strength of scars in mouse	[135]
Pulsed DC	0.9–1.9 kV/m	33.3 kpps				Remodeling	Accelerated healing in mice wound	[137]

Clinical trials (Table 6) with voltage stimulation are seen to explore pulsed DC voltages showing improvement in wound closure and area reduction for pressure ulcers [41, 138], venous leg ulcers [139], chronic wounds [140], and diabetic foot ulcers [42]. The stimulation parameters are in the range of 150 V and 6000 ppm, aligning with the parameters seen in other works.

**Table 6:** Voltage stimulation for wound healing, clinical trials

Stimulation parameters			Electrode parameters			Effect of stimulation	References
Type	Amplitude	Frequency (ppm)	Duration	Material	Dimension	Effect	
Pulsed DC	100 V	6000 ppm	50 min for 5 days/week	Carbon rubber	20 cm apart	Wound area reduction in pressure ulcers	[41]
Pulsed DC	100 V	6000 ppm	50 min for 5 days/week	Carbon rubber		Wound area reduction in pressure ulcers	[139]
Pulsed DC	50–150 V	6000 ppm	3 h/day for 90 days		4.8 × 10.2 cm^2^ active, 12.7 × 20.2 cm^2^ passive	Wound closure and area reduction of pressure ulcers	[138]
Pulsed DC	150 V	6000 ppm	45 min, 3 times/week for 28 days	Metallize gauze	20 cm apart	Wound area reduction of chronic ulcer wounds	[140]
Pulsed DC	50 V	6000 ppm	8 h for 84 days			Wound area reduction of diabetic foot ulcer	[42]
Pulsed DC	20–80 V	3600 ppm	4 times/week for 90 days		45 × 22 mm^2^	Increase angiogenic response and hemoglobin levels in acute wounds	[105]

## Disparity in Approaches

Electrical stimulation has long been used in different stages of wound healing. As discussed in Sect. “Overview of Approaches” and summarized in Tables 1, 2, 3, 4, 5, and 6, various groups have shown the effects in different stages of healing, using different types of stimulation (current, voltage), in different types of tissue models (in vitro, in vivo) and with various experimental setup. However, the results of many approaches lack repeatability due to the unavailability of experimental parameters. As biological modulation by electrical means is a complex process, the stimulus is dependent on many factors. Incomplete reporting of experiments creates ambiguity among various results.

Considering in vitro experiments, a DC stimulation around 100 mV/mm is shown to have different effects in separate experimental setups including increasing fibroblast viability [37], migration of fibroblasts [112, 118], migration of endothelial cells [61], migration of keratinocytes [38], migration of epithelial cells [127], proliferation of keratinocytes [65], and regulating growth factors [131]. Although none of the works have identical setups or comparable electrode parameters. Again, an AC stimulation around 100 mV/mm and 10–60 Hz frequency showed regulation of collagen expression and cytotoxicity in fibroblast [129]. Considering current stimulations, a pulsed current of 100 µA is reported to cause galvanotaxis of fibroblasts [78] and also modulate the growth of fibroblasts [79] for different pulse rates and experimental setups.

On the other hand, similar biological outcomes are seen for different electrical stimulation parameters. Various in vitro works reported migration of fibroblasts, proliferation of fibroblasts, transdifferentiation of fibroblasts, migration of keratinocytes, antibacterial effects, etc. Although having similar effects, none of the experiments could be compared to others due to differences in experimental setup. Some of the parameters having similar effects are summarized in Table 7 for comparison.

**Table 7:** Stimulation parameters having similar effects

Effect	Stimulus parameter	References
Migration of fibroblast	2 µA DC	[77]
	25–100 mV/mm DC	[36]
	100 mV/mm DC	[112]
	0–100 mV/mm DC	[115]
	2 V–1 Hz AC	[113]
	10 V–4 Hz AC	[121]
	0.5 V–1 Hz AC	[122]
Proliferation of fibroblasts	2 µA DC	[77]
	2–100 µA 11 ppm pulsed DC	[79]
	50, 200 mV/mm DC	[117]
	20–100 mV/mm DC	[118]
	1–5 V pulsed DC	[66]
	180 mV–1.688 Hz AC	[120]
	10 V–4 Hz AC	[121]
Antibacterial effect	0.4–400 µA DC	[72, 73]
	0.2–1 mA DC	[74]
	500 µA	[75]
	20–2000 mA	[35]
	250 V DC	[108]
	500 V DC	[107]

Analyzing the parameters shows that a particular stimulation could have multiple biological outcomes whereas the same biological effect is seen from different types of stimulation. Furthermore, without the full information of experimental setups, it would be tenuous to replicate, scale, and transfer one approach into another or propose new methods. A protocol for reporting is required to describe all the outcomes in a relatable and repeatable manner.

## Parameters Affecting Wound Healing

From the discussion in the previous sections, it is evident that although the research works resulted in specific biological alterations, the stimulation environment varies in many aspects. The experimental parameters, up to much of an extent, affect the outcome of the study. Some of the parameters affecting electrical stimulation are discussed hereby.

### Experimental Setup Parameters

The outcome of experiments, to some extent, depends on the configuration of the setup. Demir et al. used a constant DC stimulation of 300 µA using carbonized rubber electrodes on rat tissue to shorten the inflammatory phase [81], whereas the same stimulation was applied on pig tissue by Alvarez et al. using silver-coated electrodes to observe migration and proliferation of epithelial cells [91]. Although the stimulation parameters might be the same, the setup of electrodes, the distance between the electrodes, and electrode materials play a vital role in delivering the stimulus to the tissue.

#### Electrode Configuration

Electrodes are the interface between the stimulating circuitry and tissue. Hence, the placement of electrodes is important for effective delivery of stimulus. Most of the works prefer a bipolar configuration of electrodes, although variations in placement on tissue are seen.

**Fig. 7:**
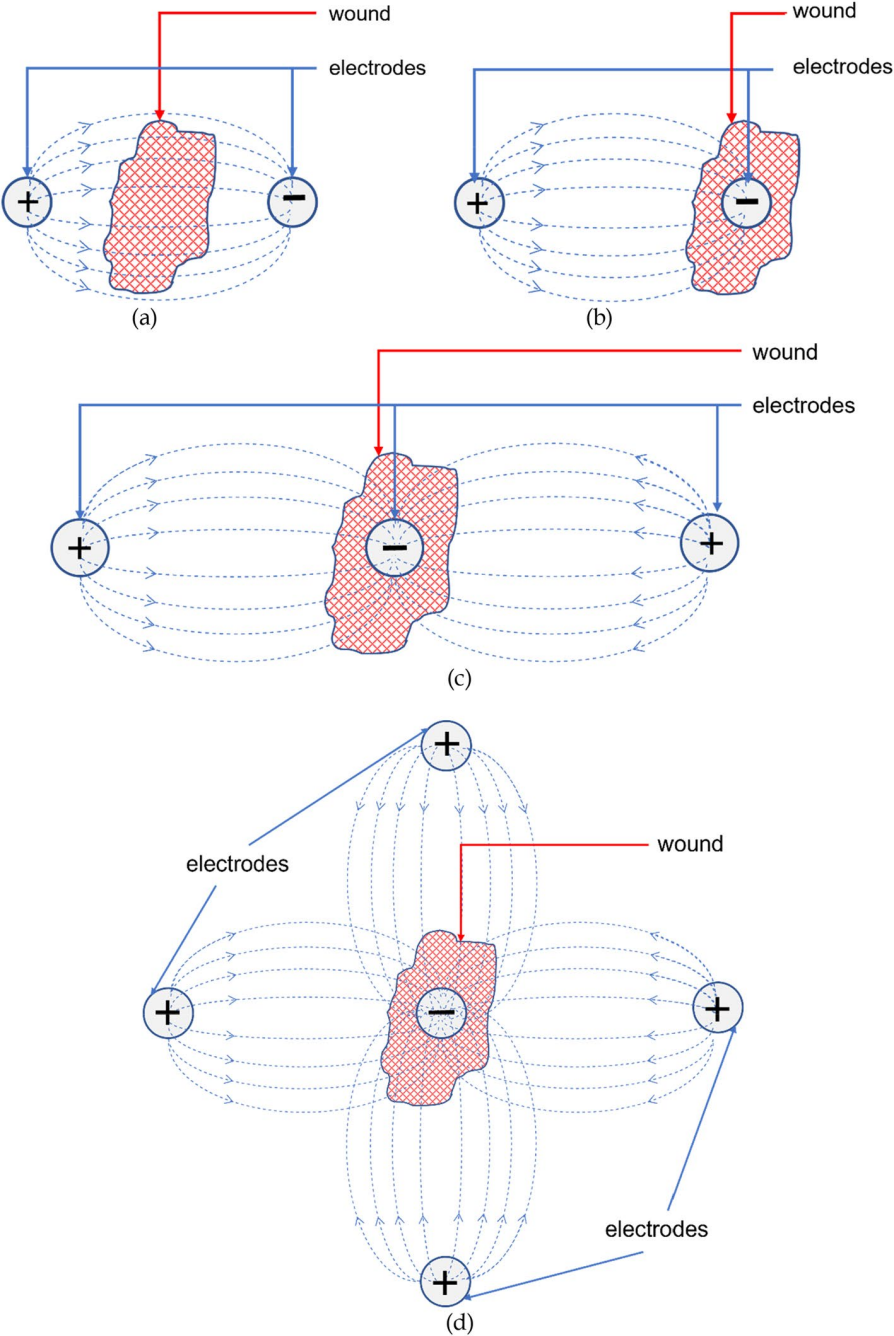
Electrode configurations. **a** Bipolar configuration with electrodes on both sides of the wound. **b** Bipolar configuration with one electrode on the wound. **c** Tripolar configuration. **d** Tetrapolar configuration

A common configuration is placing electrodes across the wound as shown in Fig. 7a. The electrodes are placed on the periphery of wounds on unwounded sites and the current flows through the wound site [83]. Although the placement as in Fig. 7b is also seen in many instances where an electrode is placed on the wound site while another is placed on an unwounded site [136]. It can be argued for the configuration of Fig. 7a that cells near the wound edge of the positive electrode get a supportive stimulation for directional movement towards the wound while the cells near the negative electrode get an opposing stimulation. This dilemma can be addressed with the configuration of Fig. 7b where only one edge of the wound is stimulated. Multi-electrode configurations have also been explored by different groups where two positive electrodes were placed on two sides of the wound with a negative electrode on the wound site, as shown in Fig. 7c, resulting in directional movement of cells [133]. A similar and more complex approach was seen where four positive electrodes were placed around the wound with one negative electrode on the wound site (Fig. 7d) [84]. A comparison between various configurations would prove to be worthy of an effective stimulation protocol.

#### Electrode Polarity

Various research works have proven the effect of external stimulation on the directional movement of cells. Therefore, the effect of polarity of stimulating electrodes in and around the wound site is eminent. Research has shown that macrophages and neutrophils involved in the inflammatory phase of wound healing have a negative polarity [141, 142]. Stimulations enhancing the inflammatory phase would require placing positive electrodes on the wound site to accelerate healing as shown by Bolton et al. [85]. Similarly, epidermal cells show negative polarity [143], and a positive polarity electrode placed on the wound enhances re-epithelialization [133]. On the other hand, keratinocytes are shown to have a positive polarity [38], and so a negative electrode on the wound site would provide support to the differentiation of keratinocytes, which is depicted in the in vivo works of Liang et al. [133]. A simulation with varying polarity corresponding to the stages of healing would provide a ground for effective stimulation.

#### Electrode Geometry

The geometry of the electrode includes the distance between electrodes and the shape of the electrodes used. Various studies have highlighted the effects of inter-electrode distance on wound healing [144, 145], as it was shown to play a vital role in directing exogenous stimulation current to the wound site to enhance the healing process. A comprehensive study demonstrated the relation between activation depth and activation volume with the size of the electrode and inter-electrodes distance [146]. Larger electrodes placed closely tend to show more activation volume compared to smaller electrodes placed at the same inter-electrode separation. That effect diminishes with larger inter-electrode separations. Electrodes of smaller surface areas were also shown to allow for deeper stimulation effects. The above indicates a significant correlation between the geometry of the electrodes and the localization of the effects of the applied stimulation through the biological medium. Gomes-Tames et al. pointed out such a correction in [146], and although that work was applicable to excitable tissue, the same conclusion can be drawn for stimulating wounds and to provide a better understanding of the impact of specific electrode designs on the effects of stimulation on specific biological features.

#### Electrode Materials

Electrodes are the interface between the external stimulation circuits and biological tissues. Hence, the materials used in designing electrodes play an important role in determining the efficacy of stimulation. Some of the fundamental aspects of selecting any material for electrodes are

1. Compatibility of the material with the tissue should be good.
2. Mechanical stability of the material on tissue should be achieved.
3. Capacity of injecting the desired charge for stimulation should be maintained.
4. Toxicity due to Faradaic interactions between electrode and tissue should be low or none.
5. Corrosion of material into tissue should be avoided.
6. Material should be stable throughout stimulation.

Merrill et al. reviewed the biocompatibility and charge storage capacity of different materials for stimulation electrodes [44]. For an effective stimulation protocol, the material of choice for the electrode should also be considered along with other parameters for electrode design.

### Stimulation Parameters

Different research groups have explored various options for electrical stimulation of wound tissues. However, some stimulation parameters should be maintained to find correlation and translation between works. Such parameters are discussed hereby.

#### Type of Stimulation

Simulations are provided mainly in the form of currents or voltages. Due to less complexity in circuitry, constant voltage stimulation is preferred by many groups. Although applying a constant voltage stimulation comes with its demerits. Applying a constant voltage would enhance the current of injury; however, it would depend on other factors as well. As tissues contain different types of cells having varying impedance, the external current through the tissue due to a constant voltage would result in varying currents in different parts of the tissue. Moreover, scar formation in the wound would result in high impedance compared to other tissues resulting in a voltage drop, whereas fluid accumulation in the wound would lower the impedance of the site causing high currents to flow through the wound. Furthermore, the presence of contact impedance in the electrode–electrolyte interface would add up to the voltage drop resulting in lower currents through the wound [147].

On the other hand, constant current sources would inject a fixed amount of current through the site regardless of the contact impedances and tissue impedances. As this current can be directly correlated with the current of injury, it is possible to link it with the physiological healing process. Hence, as a parameter of interest, constant current should be preferred as a type of stimulation. Even if a constant voltage is used as stimulation, the amount of current flowing through the biological tissue should be noted for correlation with physiology.

#### Frequency

Frequency is a parameter of interest that should be defined while describing any stimulation. Depending on the frequency, the applied stimulus is divided into DC, AC, and pulsed DC. Although the efficacy of direct current stimulation is proven in many works [36, 72], it is also associated with DC blocking affecting cellular ion channels and membrane polarization [148]. Moreover, the capacitive elements of cells behave as high impedances under DC stimulation eventually being uninvolved in current transport. On the other hand, AC stimulation is seen to have different effects on healing mechanisms [89, 110]. An upside of AC stimulation in wound healing is that most of the works reported a frequency in the Hz range whereas side effects like AC blocking occur in the kHz range [149]. However, a downside of AC stimulation is the bidirectional current flow through the wound site. Quite a few groups have reported using pulsed DC stimulation for wound healing, both in the form of monophasic or biphasic waves. The stimulus is considered as pulses and measured in pulses per minute (ppm) or pulses per second (pps). Pulsed DC stimulations provide a middle ground between DC and AC stimulations, benefiting from directional current flow through the wound without causing DC blocking to the tissues.

## Stimulation Protocol for Wound Healing

Electrical stimulation has been explored for wound healing by different groups over the past decade. Despite the efforts throughout the years, a unified protocol for electrical stimulation targeted to wound healing mechanisms is yet to be proposed. Such a protocol could justify the applied stimulation and relate it to the physiological mechanism of healing. It would also provide a ground to replicate the same results and provide scalability of stimulus for different sizes of wounds.

As per the discussion in Sect. “Parameters Affecting Wound Healing,” it is evident that the value of stimulus is not enough to report an approach for wound healing. Rather the mechanism is dependent on different parameters related to the setup of the experiment. All such parameters affecting the outcome of electrical stimulation could be included to propose a protocol. For in vitro cases, the protocol can be represented as follows:

- Setup parameters
  1. *Electrode separation* this refers to the minimum distance in the conductive pathway between the stimulating electrodes.
  2. *Electrode configuration* number of electrodes used and their placement around the sample.
  3. *Electrode polarity* placement of active (anode) and passive (cathode) electrodes.
  4. *Electrode geometry* the dimensions of each electrode and the surface area in contact with the sample.
  5. *Electrode material* the material used to fabricate electrodes along with their biocompatibility and contact potentials.
  6. *Dimension of setup* this refers to the three-dimensional measurement of the setup where the biological sample is stimulated including the effective conductive area of the sample.
- Stimulation parameters
  1. *Type of stimulation* this refers to either electric field across or current density through the sample.
  2. *Frequency and pulse* this refers to the frequency of the applied stimulus. In the case of pulsed DC stimulation, the duty cycle, pulse width, and pulse duration should also be included.
  3. *Duration* this refers to the total duration of exposure.
- Sample parameters
  1. *Biological cells* the concentration, cell counts, and percentage of confluence should be mentioned.
  2. *Growth media* the constituent of the supporting growth media, concentration of the media, and growth factors involved should be mentioned.
  3. *Temperature and humidity* the temperature and humidity of the sample in which experiments are performed should be noted.

## Discussion

Electrical stimulation is reported to have various cellular effects, ranging from proliferation, transdifferentiation, and galvanotaxis to cell death and antibacterial effects depending on the applied stimulation and exposure time. Various research works reported specific biological alterations due to applied electric stimulation. However, it is tough to replicate a specific experiment due to a lack of setup parameters. The stimulation setups vary widely in many terms. As a result, the same biological outcome is proven for different setups, whereas different biological alterations for different cell types are also seen for similar stimulations.

The biological medium acts as a bulk conductor of electricity where the principal mode of conduction is through ions, whereas the external stimulating circuitry injects current involving the flow of electrons. This complex mode of charge transfer makes the physiological interaction of external stimuli dependent on various parameters. The absence of all the underlying parameters in reporting creates ambiguity and poses a problem with replicability. For example, an increase in the growth of fibroblasts is reported using 20–100 mV/mm DC stimulus [118], 1–5 V 4800 pps pulsed DC stimulus [66], and 180 mV 1.688 Hz AC stimulus [120] with completely different setups. To make results viable, such underlying parameters should be reported thoroughly and in a formatted manner.

A solution to this disparity could be to mention specific parameters of the experiment. Such a set of parameters could be considered as a protocol of interest for proposing electrical stimulation methodology. As mentioned in Sect. “Stimulation Protocol for Wound Healing,” the protocol would include parameters related to the experimental setup, the stimulus applied, and the biological sample on which the stimulation is applied. The works of Snyder et al. are a good example of mentioning all the relevant information required for replicating an experiment [36]. In terms of the protocol, the work can be formatted as follows:

- Setup parameters
  1. *Electrode separation:* 50 mm.
  2. *Electrode configuration:* bipolar.
  3. *Electrode polarity:* on both sides of the sample.
  4. *Electrode geometry:* 4.83 mm diameter glass tubes.
  5. *Electrode material*: 1% agarose solution dissolved in PBS; electric fields applied through Ag–AgCl electrodes.
  6. *Dimension of setup:* Nunclon Delta treated 4-well rectangular lid, dimensions $128 \times 86$ mm [150].
- Stimulation parameters
  1. *Type of stimulation:* 25–100 mV/mm electric field.
  2. *Frequency and pulse:* 0 Hz (DC), continuous stimulation.
  3. *Duration:* 10 min.
- Sample parameters
  1. *Biological cells:* human dermal fibroblasts cultured until confluent.
  2. *Growth media:* L-15 medium containing 4.6 g/L d-glucose and 10% fetal bovine serum.
  3. *Temperature and humidity:* 37 °C and room conditions.

Reporting the parameters according to the protocol, it is evident that all the information regarding replicating the experiment is available and it could be expected to obtain similar outcomes. Moreover, the effect of the same stimulus on a different cell line can be compared keeping the same experimental setup.

Another disparity arises from the lack of scalability of the results. Different groups use various setups to comment on the effect of electrical stimulation on a particular biological phenomenon. However, the outcome becomes trivial if it cannot be translated and scaled to a different experimental setup. In the works of Szuminsky et al., a stimulation of 500 V was applied across a separation of 3 cm [107]. Any other work referring to this literature could scale the applied voltage according to their designed electrode separation. A better approach to reporting the type of stimulation is to mention the electric field or current density of the applied stimulation. The applied constant voltage is across the sample and depends on the distance between electrodes. On the other hand, the effective current flow path in applying a constant current through the sample depends on the dimensions of the sample. For a scalable quantity regardless of the sample size, the constant voltage could be translated into an electric field across the sample, while the constant current could be replaced by the current density through the sample along with the setup parameters mentioned in the protocol.

Although the proposed protocol does give a good hold over the replicability and scalability of the in vitro experiments, the question of transferability and translation between experiments remains. Transferability comes from the fact that the same outcome of an experiment can be achieved regardless of applying the stimulation in the form of current or voltage. There should be a conversion factor for transferring stimulus from voltage to current and vice versa. The measured impedance of the sample could be considered as a parameter for conversion. However, conduction through the biological medium is a three-dimensional mechanism involving all possible pathways of conduction between the electrodes. Hence the measured impedance (*Z*) is dependent on the setup parameters as follows:

$$Z= \zeta \frac{L}{A},$$

where *L* is the effective conduction length between the electrodes and *A* is the cross-sectional area of the sample under consideration. Here $\zeta$ is the measured impedivity of the biological sample. Impedivity reflects the impedance per unit length and unit cross-sectional area and is determined by the electric and dielectric properties of the sample [151]. Biological cells are in general modeled as a complex impedance consisting of a network formed of resistance and capacitance [152]. Any external stimulation would produce a frequency-dependent impedance. Impedivity depends on the concentration of cells, capacitance of the cell membranes, conductivity of the interstitial fluid, and the intracellular medium [153]. All these parameters along with impedivity could be considered for transferability among experiments. Furthermore, translation of in vitro to in vivo works can also be proposed using impedivity. As tissues consist of different types of cells, each of them having their impedivity, the applied stimulus could be tailored to address and focus each type of cell during the whole process of wound healing.

**Fig. 8:**
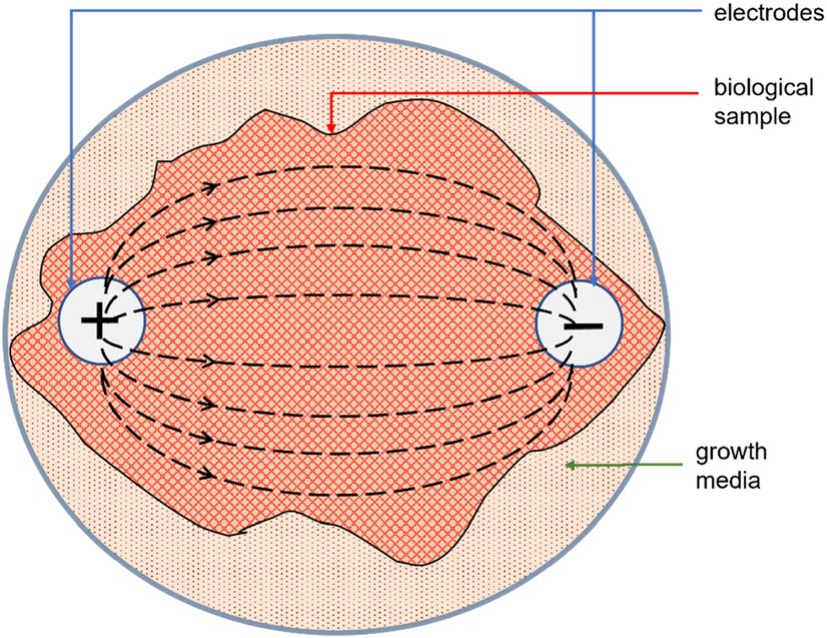
Conduction pathways through sample (top view)

**Fig. 9:**
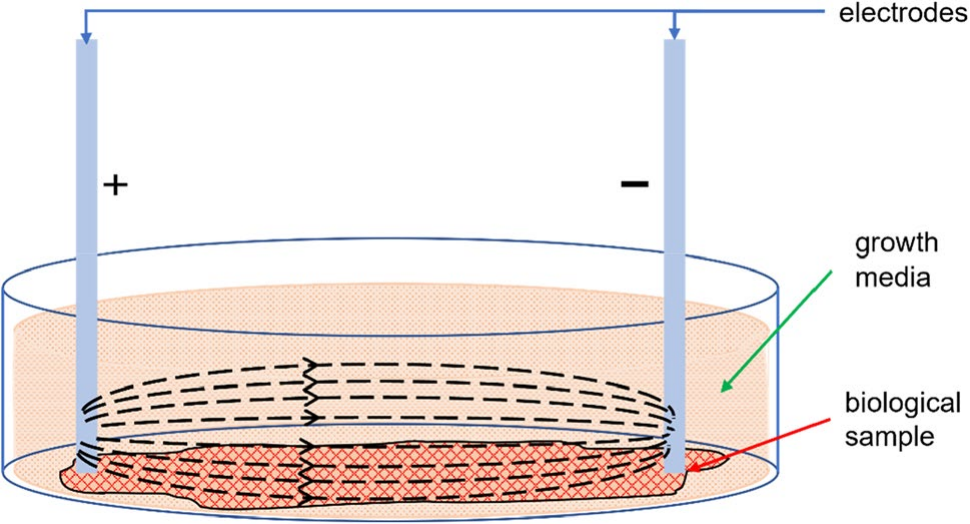
Conduction pathways through sample (side view)

Nevertheless, impedivity cannot be considered as the complete solution. In in vitro studies, cells are suspended in a growth medium. The applied stimulus would generate a current pathway through the cells as well as through the medium. The current pathways may look as in Fig. 8. But considering the side view, the pathways may look like Fig. 9. The relative positioning of the electrodes to the sample determines the amount of current flowing through the cells suspended at the bottom of the culture plate. Further exploration is required to find the optimum positioning of electrodes and the contribution of cell impedivity towards the total measured impedivity of the sample.

## Conclusion

Natural wound healing of the skin is a lengthy process that requires adequate coordination between various factors. Any deviation in the process leads to incomplete or varied healing resulting in the accumulation of fluids and scar tissue. Moreover, this time-staking process takes a toll on the quality of life of the patients. Hence, any external approach to accelerate and control the healing process is taken positively.

Electrical stimulation in therapeutic view towards wound healing has been reported since the mid-twentieth century. Several works proved that it is a useful modality in treating wound healing. In vitro and in vivo research paved the way for clinical trials to establish electrical stimulation as an acceptable treatment method. Even though the effects of this modality in enhancing wound healing are widely circulated, electrical stimulation has not yet been accepted as an established mode of treatment. The lack of convergence among the research outcomes is pointed out as a reason. A guideline to report experimental works is a way of unification among the outcomes of different research. It would remove discrepancies, give complete information about the experiment procedure, and create a standard process to compare different approaches. Such a guiding protocol would resolve the problem of repeating experiments in different environments and also remove the dependence of the stimulus on the experimental setup. It would also help in finding the optimum stimulation for particular types of cells without causing any secondary harm to other excitable or non-excitable cells.

An interesting outcome would be a unified protocol for conducting electrical stimulation experiments including in vitro, in vivo, ex vivo, and clinical cases. The unified protocol would be an extension and translation of the proposed protocol for in vitro experiments. Although this would involve the inclusion of new parameters of interest. As the biological entities involved in in vivo, ex vivo, and clinical cases are complex tissues involving multiple types of cell lines, the stimulation for one type of cell line might cause diverse effects on other types of cell lines in the tissue. A cross-analysis of the results from systematic experimentation of multiple cells in vitro and tissues in vivo would provide a good idea about the unified protocol for electrical stimulation in wound healing.

Although electrical stimulation has been used widely in experiments regarding both excitable and non-excitable tissue, the secondary effects of stimulation should also be taken into consideration. One of the prominent secondary effects of electrical stimulation is tissue damage. Early works by McCreery et al. suggested the use of Shannon’s equation to estimate the threshold of electrical stimulation to prevent tissue damage during therapeutics [154]. A comprehensive review in this aspect has been done by Cogan et al. where they report the role of pulse frequency, duty cycle, current density, and electrode size in causing tissue damage during stimulation [155]. Tissue damage due to current densities of 50 µA/mm^2^ and 50 Hz has been reported during intramuscular electric stimulation [156]. Another notable secondary effect of electrical stimulation is the generation of free radicals. For example, stimulation waveforms featuring 9 V amplitude, 1 ms pulse width, and 4 Hz frequency have been reported to generate free radicals which oxidize drugs in the system and cause inhibitory effects [157]. Free radical production is also reported in macrophages when stimulated with 50 Hz electric fields [158]. Such secondary effects cause deviation in the expected outcome of the stimulation. In in vitro and ex vivo cases, the proper stimulation threshold should be considered to avoid tissue damage. In stimulating non-excitable cells in vivo, proper measures should be taken to reduce the effect on excitable cells as well as to minimize tissue damage.

A good grasp on the controlling mechanisms of healing by electrical influence could pave the way to design stimulators for complex tissue models. Varying the stimulation parameters would allow for maximum stimulation at deeper tissues or parts of the tissues at a time. It could also help in decelerating any specific process of healing for the sake of uniform and homogeneous healing. Modulating the process of natural healing through electrical stimulation would bolster establishing it as a proven clinical modality of treatment.

## Data Availability

Not applicable.
